# A Quadruple Knockout of *lasIR* and *rhlIR* of *Pseudomonas aeruginosa* PAO1 That Retains Wild-Type Twitching Motility Has Equivalent Infectivity and Persistence to PAO1 in a Mouse Model of Lung Infection

**DOI:** 10.1371/journal.pone.0060973

**Published:** 2013-04-10

**Authors:** James J. Lazenby, Phoebe E. Griffin, Jennelle Kyd, Cynthia B. Whitchurch, Margaret A. Cooley

**Affiliations:** 1 School of Biotechnology and Biomolecular Sciences, University of New South Wales, Sydney, Australia; 2 School of Medicine, University of Tasmania, Hobart, Australia; 3 CQUniversity, Rockhampton, Australia; 4 ithree institute, University of Technology, Sydney, Sydney, Australia; The Scripps Research Institute and Sorrento Therapeutics, Inc., United States of America

## Abstract

It has been widely reported that quorum-sensing incapable strains of *Pseudomonas aeruginosa* are less virulent than wild type strains. However, quorum sensing mutants of *P. aeruginosa* have been shown to develop other spontaneous mutations under prolonged culture conditions, and one of the phenotypes of *P. aeruginosa* that is frequently affected by this phenomenon is type IV pili-dependent motility, referred to as twitching motility. As twitching motility has been reported to be important for adhesion and colonisation, we aimed to generate a quorum-sensing knockout for which the heritage was recorded and the virulence factor production in areas unrelated to quorum sensing was known to be intact. We created a *lasIRrhlIR* quadruple knockout in PAO1 using a published technique that allows for the deletion of antibiotic resistance cartridges following mutagenesis, to create an unmarked QS knockout of PAO1, thereby avoiding the need for use of antibiotics in culturing, which can have subtle effects on bacterial phenotype. We phenotyped this mutant demonstrating that it produced reduced levels of protease and elastase, barely detectable levels of pyoverdin and undetectable levels of the quorum sensing signal molecules N-3-oxododecanoly-L-homoserine lactone and N-butyryl homoserine lactone, but retained full twitching motility. We then used a mouse model of acute lung infection with *P. aeruginosa* to demonstrate that the *lasIRrhlIR* knockout strain showed equal persistence to wild type parental PAO1, induced equal or greater neutrophil infiltration to the lungs, and induced similar levels of expression of inflammatory cytokines in the lungs and similar antibody responses, both in terms of magnitude and isotype. Our results suggest, in contrast to previous reports, that lack of quorum sensing alone does not significantly affect the immunogenicity, infectiveness and persistence of *P. aeruginosa* in a mouse model of acute lung infection.

## Introduction

The role of quorum sensing (QS) and biofilm formation in *Pseudomonas aeruginosa* biology is clear, and previous reports have suggested that knocking out QS systems in *P. aeruginosa* results in decreased virulence in animal models of infection [1], [2], [3], [4], [5] and has impacts on bacterial growth rate in culture. The deletion of both the synthase genes and the responder genes for both 3-oxo-N-dodecanoyl-L-homoserine lactone (3OC12HSL) and the other principal homoserine lactone QS signal produced by *P. aeruginosa*, N-butyryl-L-homoserine lactone (C4HSL) should result in a quadruple knockout that will not respond to or produce 3OC12HSL or C4HSL. Previously reported *lasI/rhlI* knockouts including PAO1-JP2 have been reported to be less virulent than wild type *P. aeruginosa* PAO1 in a range of animal models [3], [4], [5], [6], [7], [8], [9], [10], [11]. However, QS mutants of *P. aeruginosa* have been shown to develop spontaneous mutations under prolonged culture conditions [12], [13], and one of the phenotypes of *P. aeruginosa* that is frequently affected by this phenomenon is type IV pili-dependent motility, referred to as twitching motility [12]. In fact, twitching motility was originally reported to be controlled by QS [14] but was later confirmed to be independently regulated [15]. Interestingly, JP-2 has been reported to be incapable of twitching motility [14] which suggests that this strain is likely to contain secondary mutations in a gene (or genes) required for twitching motility. It has been shown that, unlike the PAO1 parental strain, a PAO1 mutant with defective pili is unable to bind to respiratory epithelial cells and cannot induce production of the proinflammatory and neutrophil-recruiting chemokine interleukin (IL)-8 [16]. It has also been reported in many studies that type-IV pili are integral for adherence to and colonization of mucosal surfaces (reviewed in Hahn *et al.* [17]). It is therefore important that twitching motility remains intact in any QS knockout *P. aeruginosa* used for *in vivo* infection studies assessing the role of QS in bacterial infectivity and persistence.

Because of the inherent and spontaneous mutations that occur during standard laboratory subculturing of *P. aeruginosa*, we created a *lasIRrhlIR* knockout in a strain for which the heritage was recorded and the virulence factor production in areas unrelated to QS was known to be intact. We used a technique described by Hoang *et al.* [18], which allows for the deletion of antibiotic resistance cartridges following mutagenesis, to create an unmarked QS knockout of PAO1. Such a knockout strain has two benefits: firstly an unmarked knockout can be further genetically modified without the need for multiple resistance cassettes and secondly, it has been found that subinhibitory concentrations of antibiotics can profoundly influence the phenotype of *P. aeruginosa* [19], [20], [21]. A knockout strain that does not require antibiotic selection for maintenance of mutation would allow the comparison of QS knockout strains to isogenic wild-type bacteria without any possibility of phenotypic changes being generated in the bacteria because of the presence of antibiotics in the culture medium.

After creation of the quadruple *lasIRrhlIR* knockout, we tested it in a mouse model of *P. aeruginosa* lung infection to compare the course of infection with that of the parental PAO1. We found that the knockout showed equal persistence, induced comparable or greater inflammation, and resulted in identical antibody responses in terms of magnitude and immunoglobulin isotype to parental PAO1.

## Materials and Methods

### Bacterial Strains, Plasmids and Media

The wild type *P. aeruginosa* used as the basis for this study was PAO1 strain ATCC 15692. *E. coli* strain DH5α was used in all genetic manipulations and in the preparation of DNA sequencing templates, and *E. coli* S17-1 was used as the donor strain in bacterial conjugation for allelic exchange mutagenesis. All bacterial strains used in this study are listed in Table 1.

**Table 1:** Bacterial strains and plasmids used in this study.

Bacterial strains	Relevant characteristic(s)	Source or reference
*E. coli*		
DH5α	*recA endA1 gyrA96 hsd*R17 *thi-1 supE44 relA1* φ80 d*lacZ*Δ*M15*	Invitrogen
S17-1	*thi pro hsdR recA chr::RP4-2*	[44]
*P. aeruginosa*		
PAO1	Wild type *P. aeruginosa* strain ATCC 15692	American Type Culture Collection
PAO1Δ*lasIR*	PAO1 with deletion of *lasIR* region	This study
PAO1Δ*rhlIR*	PAO1 with deletion of *rhlIR* region	This study
PAO1Δ*lasIRrhlIR*	PAO1Δ*rhlIR* with deletion of *lasIR* region	This study
**Plasmids**		
pPS854	FRT cassette vector	[18]
EZ-TN5<TET-1>	Source of Tet^R^ gene	Epicenter
		
pGEM-T	*E. coli* cloning vector; Ap^R^	Promega
pOK12	*E. coli* cloning vector; Km^R^	[45]
pRIC380	*P. aeruginosa* suicide vector; Ap^R^	[46]
pFLP2		[18]
pSB406	C4HSL biosensor plasmid	[23]
pSB1073	3OC12HSL biosensor plasmid	[24]
pCBW108	pPS854 containing 1.4 Kb Tet^R^ gene amplified from EZ-TN5<TET-1> cloned into *Eco*RI site.Source of FRT/Tet^R^ cassette	[47] This study
pJL001	1 Kb *las*R upstream region amplified from PAO1 genome in pGEMTeasy; Ap^R^	This study
pJL002	1 Kb *las*I downstream region amplified from PAO1 genome in pGEMTeasy; Ap^R^	This study
pJL003	1 Kb *rhl*R upstream region amplified from PAO1 genome in pGEMTeasy; Ap^R^	This study
pJL004	1 Kb *rhl*I downstream region amplified from PAO1 genome in pGEMTeasy; Ap^R^	This study
pJL005	pOK12 containing *las*R upstream region and *las*I downstream region ligated at *Sac*I sitesand cloned into *Xho*I and *Nde*I sites of pOK12; Km^R^	This study
pJL006	pOK12 containing *rhl*R upstream region and *rhl*I downstream region ligated at *Sac*I sitesand cloned into *Kpn*I and *Nde*I sites of pOK12; Km^R^	This study
pJL007	pJL005 containing 1.4 KB FRT/Tet^R^ cassette from pCBW108 cloned into *Sac*I site; Km^R^, Tc^R^	This study
pJL008	pJL006 containing 1.4 KB FRT/Tet^R^ cassette from pCBW108 cloned into *Sac*I site; Km^R^, Tc^R^	This study
pJL009	3.4 Kb *Spe*I insert from pJL008 cloned into pRIC380; Ap^R^, Tc^R^	This study
pJL010	3.4 Kb *Spe*I insert from pJL009 cloned into pRIC380; Ap^R^, Tc^R^	This study

*P. aeruginosa* and *E. coli* were cultured in LB-Lennox broth (LB) or cation-adjusted Mueller Hinton broth (CAMHB) or on LB solidified with 1.5% agar (LBA). Antibiotic concentrations used for selection of *E. coli* were 100 µg/mL ampicillin, 12.5 µg/mL tetracycline, and 50 µg/mL kanamycin and for *P. aeruginosa* were 250 µg/mL carbenicillin, and 200 µg/mL tetracycline.

### Construction of PAO1Δ*lasIR*, PAO1Δ*rhlIR, and* PAO1Δ*lasIRrhlIR*

Unmarked deletion mutants of PAO1 were constructed using the Flp-FRT recombination system for site specific excision of chromosomal sequences described [18]. Briefly, 1 kb sections that flanked the *lasIR* and *rhlIR* region(s) to be deleted from PAO1 chromosomal DNA were PCR amplified and cloned into pGEMTeasy and sequenced to ensure that no mutations in these flanking regions had been introduced by PCR. The upstream and downstream flanking regions of the *las* or *rhl* regions were ligated and then cloned into pOK12 (creating pJL005 and pJL006 respectively) and the *FRT*/Tet^R^ cassette from pCBW108 cloned into the internal *Sac*I site resulting in pOK12 constructs pJL007 and pJL008 containing the flanking regions of *lasIR* or *rhlIR* regions separated by the *FRT*::Tet^R^ cassette. The resultant clones were then digested with *Spe*I and cloned into the suicide vector pRIC380. This vector carries the genes *sacBR,* which promote sensitivity to sucrose, and *oriT* which enables conjugal transfer. The resultant clones were transformed into the *E. coli* donor strain S17-1 in preparation for mating with *P. aeruginosa* PAO1. Following conjugation, the transconjugants were plated onto LBA without sodium chloride and supplemented with 5% sucrose and containing tetracycline to select for colonies in which the plasmid had excised while leaving the homologously recombined *lasIR*::Tet^R^ or *rhlIR*::Tet^R^ alleles in the chromosome. The Tet^R^ gene was then excised using the pFLP2 plasmid that expresses the Flp recombinase as described previously [18] creating *P. aeruginosa* strains with the *lasIR* or *rhlIR* regions deleted and replaced with an FRT sequence. Allelic exchange deletion mutants were confirmed by both PCR and Southern hybridization of isolated chromosomal DNA. A list of all plasmids used in this study is given in Table 1 and all primers used are listed in Table 2.

**Table 2:** Primers used in this study.

Oligonucleotide	Sequence 5′−3′	Description
*TetR* Forward	GCG*GAATTC*ACCTGAAGTCAGCCCCATACG	*Eco*RI site inserted at 5′ end (italics)
*TetR* Reverse	CGC*GAATTC*CTGCCAAGGGTTGGTTTGC	*Eco*RI site inserted at 5′ end (italics)
*lasR* Upstream Flanking Region Forward	*CTCGAG* GCGTAGCGATGGGCAACAAG	*Xho*I site inserted at 5′ end (italics).
*lasR* Upstream Flanking Region Reverse	*GAGCTC* CGTAGTCCTGGCTGTCCTTAGGC	*Sac*I site inserted at 5′ end (italics).
*lasI* Downstream Flanking Region Forward	*GAGCTC* AACAGCGACTGGCGGTTTC	*Sac*I site inserted at the 5′ end (italics)
*lasI* Downstream Flanking Region Reverse	*CATATG* GCCTGAACGACCTGAACGG	*Nde*I site inserted at the 5′ end (italics).
*rhlR* upstream Flanking Region Forward	*GGTACC* GCACGCATCGCTCACGAG	*Kpn*I site inserted at the 5′ end (italics)
*rhlR* Upstream Flanking Region Reverse	*GAGCTC* CGTCCCACCACAGCAAAAAG	*Sac*I site inserted at the 5′ end (italics)
*rhlI* Downstream Flanking Region Forward	*GAGCTC* GCCGCAGAAGGTCAAGGG	*Sac*I site inserted at the 5′ end (italics)
*rhlI* Downstream Flanking Region Reverse	*CATATG* CCTCGCCGCCCCTGTATTAC	*Nde*I site inserted at the 5′ end (italics)

### Assays of Bacterial Phenotypes

#### Growth curves

*P. aeruginosa* strains were cultured overnight in CAMHB at 37°C shaking at 250 r.p.m. Quadruplicate 200 µL aliquots of 1∶100 dilutions of overnight cultures were added to wells of a sterile 96 well plate (BD Biosciences, Mountainview, CA), which was then incubated for 10 h at 37°C shaking in a BIO-TEK® Synergy™ HT microplate luminometer (BIO-TEK, Winooski, VT, USA). Optical density readings (605 nm) were taken every 20 min and subtracted from the blank (CAMHB only).

#### Extraction of acylated homoserine lactones (AHLs) from culture supernatant and AHL bioluminescence assay

The supernatant of 8 h cultures of *P. aeruginosa* strains cultured in LB at 37°C shaking at 250 r.p.m. was collected by centrifuging the culture at 3000× *g* for 10 min. The supernatant was sterilised though a 0.22 µm filter and AHLs extracted using a method adapted from that described by Rice *et al.* [22]. Equal volumes of culture supernatant and 0.01% glacial acetic acid in ethyl acetate were mixed, centrifuged at 10000× *g* for 2 min and the organic layer containing the AHL retained. This was repeated at least three times and the collected organic phase dried overnight at room temperature. The sediment was resuspended in 50 µL of ethyl acetate.

3OC12HSL and C4HSL were quantified using bioluminescence reporter strains as described previously [23], [24]. Briefly, 20 µL of culture supernatant or AHL standards were added to the wells of a white opaque microtiter plate (PerkinElmer, Wellesley, MA, USA), and 180 µL of mid-log phase cultures of *E. coli* containing pSB1073 or pSB406 was added to the microtiter plate, gently agitated and incubated at room temperature for 1 h, after which the luminescence was measured using a Veritas Microplate Luminometer (Veritas, Sunnyvale, CA) or a BIO-TEK® Synergy™ HT microplate luminometer (BIO-TEK). The concentration of AHL in culture supernatants was determined using dilutions of synthetic AHL as a standard curve. The results were standardized to the expression from a vehicle control (the limit of detection for the assay), and because the interassay variation was large, the results are presented as a percentage of the wild-type.

#### Twitching motility assay

Twitching motility was assayed using the subsurface stab assay described previously [25]. Briefly, the *P. aeruginosa* strain to be tested was stab inoculated through a plate of LB solidified with 1% agar to the underlying Petri dish and incubated at 37°C with saturated humidity. After 20 h at 37°C the diameter of the interstitial biofilm was measured.

#### Rhamnolipid synthesis assay

Triplicate 5 µL aliquots of overnight culture were applied to a dry rhamnolipid assay agar (prepared as described by Kohler *et al.* [26]) and allowed to dry. The plate was incubated at 37°C for 18 h then chilled overnight at 4°C. The plates were then examined for the presence or absence of a zone of clearance.

#### Skim milk protease assay

This method was adapted from that described by Sokol *et al.* [27]. In brief, three 10 µL droplets of overnight broth culture were applied to a dry skim milk agar plate and grown for 18 h at 37°C. The zone of clearance was measured with the diameter of the colony subtracted.

#### Elastase assay

The determination of elastolytic activity of 16 h *P. aeruginosa* cultures was performed using a protocol based on those described by Ohman *et al.* [28] and Rust *et al.* [29]. In brief, *P. aeruginosa* strains were cultured in CAMHB and then centrifuged at 3000× *g* for 10 min. The supernatant was then filter sterilized using a 0.22 µm syringe filter and either used immediately or stored at 4°C for up to 24 h. In triplicate, 10 mg of elastin Congo red (ECR), 500 µL of ECR buffer (0.1 M TrisCl and 10 mM CaCl_2_) and either 500 µL of the sterile spent supernatants or 500 µL of a CAMHB negative control were added to 5 mL polystyrene tubes (BD Falcon®, BD Biosciences) and incubated at 37°C for 6 h with 250 r.p.m. shaking. The incubation was stopped by the addition of 100 µL of 0.12 M Na_2_EDTA and the remaining solids were pelleted by centrifugation at 3000× *g* for 10 min. The soluble Congo red in the aqueous supernatant was quantitated using duplicates of each supernatant in a 96-well microtiter plate (BD Biosciences) with the CAMHB negative control as a blank and measuring the absorbance at 495 nm in a Biomek spectrophotometer (Beckman Coulter, La Brea, CA, USA).

#### Pyocyanin extraction and assay

The pyocyanin production from 16 h cultures of *P. aeruginosa* was determined by first extracting the pyocyanin from the culture supernatant as described by Hassett *et al.* [30]. To assay pyocyanin, duplicate 100 µL aliquots of the HCl extraction were added to microtiter wells and the absorbance at 520 nm was measured in a Biomek spectrophotometer (Beckman Coulter).

### Mouse Lung Infection Models

#### Ethics statement

All mouse studies were conducted in accord with the Australian Code of Practice for the Care and Use of Animals for Scientific Purposes, and were approved by the Animal Experimentation and Ethics committees of CQUniversity (approval No. A09/02-242) and University of New South Wales (approval Nos. 04/105A and 03/10). Animals were anesthetized for infection as described below, and were euthanized for tissue collection. All experiments were designed to use the minimum number of animals consistent with statistical validity, and all animals were monitored daily and euthanized if signs of distress were detected.

#### Acute infection model

For studies of acute infection and inflammation, groups of five male BALB/c mice 8–12 weeks old (ARC, Perth, WA, Australia) were inoculated intratracheally at day 0 with 1×10^6^ colony forming units CFU) of PAO1 or PAO1*ΔlasIRrhlIR* in 20 µL PBS containing 0.03% ethanol. A group of mice were also sham treated (anesthetized and cannula inserted, but no treatment given). Mice were sedated with a tail vein injection of Alfaxan at 12 mg/kg body weight (90 mg alphaxalone, 30 mg alphadolone acetate; Schering-Plough Animal Health, Baulkham Hills, NSW, Australia), and treatment administered by means of a 20 G paediatric cannula (Terumo Medical, Macquarie Park, NSW, Australia) inserted in the trachea. Mice from all treatment groups were then sacrificed at 4 h, 24 h or 10 days after infection, and lung tissue and BAL collected for analysis. For analysis of antibody responses, groups of five mice were given 1×10^3^ CFU of PAO1 or PAO1*ΔlasIRrhlIR* in a 0.5% agar slurry administered intratracheally by the method described above. These mice were bled by cardiac puncture under terminal anesthesia at day 21. Blood was collected in a 1.1 mL Z-gel tube (Sarstedt, Mawson Lakes, SA, Australia) that was then left at room temperature for at least 30 min before centrifuging at 2000× *g* for 10 min. The serum was collected and kept frozen at −20°C until use for evaluation of anti-Pseudomonas serum antibody concentrations.

#### Bronchoalveolar lavage (BAL)

To obtain BAL, 0.5 mL of sterile PBS was slowly flushed into the lungs of euthanized mice, and as much as possible of the PBS was recovered. Differential cell counts were performed on cytospin preparations of the BAL fixed with DPX mountant (Scharlau, Barcelona, Spain) and stained using the Diff-Quik Staining Kit 64851 (a modified version of the Wright’s stain; Veterinary Medical Surgical Supply, Maryville, NSW, Australia) according to the manufacturer’s instructions.

#### Bacterial enumeration in the lung tissue and BAL

A section of the mouse lung was homogenized in 2 mL sterile PBS using a tissue homogenizer. The bacterial CFU in the BAL fluid and homogenized lung were determined by making serial dilutions and plating onto LBA.

#### Preparation of cDNA from mouse lung tissue

Total RNA was extracted from the lung tissue using TRIzol® Reagent (Invitrogen, Mulgrave, VIC, Australia) with a maximum of 100 mg of tissue per mL of TRIzol® Reagent. Tissue was removed from RNAlater® storage solution and homogenized by bead dissociation in TRIzol® using 2.4 mm zirconia/silica beads (Daintree Scientific, St Helens, TAS, Australia). Beads and cellular debris were pelleted by centrifugation at 10 000× *g* for 10 min at 4°C. Approximately 1 mL of supernatant was removed to a fresh tube and total RNA extracted as per the TRIzol® protocol. Isolated RNA was resuspended in 50 µL of DEPC-treated water (Ambion) and DNAse treated using a TURBO DNA-free kit™ (Ambion) according to the manufacturer’s instructions. The concentration and purity of recovered RNA was quantified using a NanoDrop™ 1000 spectrophotometer (Thermo Scientific, Scoresby, VIC, Australia). cDNA was synthesized from 500 ng of total RNA using the Superscript™ III Reverse Transcriptase kit according to the manufacturer’s instructions.

#### Quantitative reverse transcription real time PCR (RT-qPCR) for inflammatory markers in mouse lung

Primer sequences for reference gene β-actin [31], TNFα, IL1β (both [32]) and IL6 ([33]) have been previously published. Primer sequences for mouse keratinocyte cytokine (KC) (analogue of human IL-8) (forward 5′–GCTGGGATTCACCTCAAGAA–3′, reverse 5′–AGGTGCCATCAGAGCAGTCT–3′) were designed from GenBank sequences using Primer 3-web version 0.3.0 [34]. Primer pairs were checked for reaction efficiency using triplicate serial dilutions of template cDNA with efficiency calculated as described in Pfaffl, 2001 [35]. Quantitative PCR (qPCR) was performed using the Platinum®SYBR®Green qPCR Supermix-UDG (Invitrogen). Each qPCR reaction contained 5 µL of cDNA diluted 1∶5–1∶10 with DEPC-treated H_2_O, and final primer concentrations of 300 nM. The reactions were performed on a RotorGene 3000® (Corbett Life Science) with an initial incubation of 50°C for 10 min then 95°C for 10 min followed by 40 reaction cycles of 95°C for 15 sec, 60°C for 30 sec, and acquiring fluorescence at 72°C for 20 sec. All products underwent melt curve analysis. Replicates showing errors were omitted from further analysis. The average of the technical replicates was used to determine the ratio of the target to the reference genes (β-actin and GAPDH) and corrected for the primer efficiency as described by Pfaffl [35]. Interassay variation was calculated by comparing the Ct values of a standard incorporated into all qPCR reactions. A coefficient of variation was calculated using PRISM® v.5 software (Graph Pad Inc. La Jolla, CA, USA) and values below 10% deemed to be acceptable.

### Detection of anti-*P. aeruginosa* Antibodies by Enzyme-linked Immunosorbent Assay (ELISA)

To prepare PAO1 sonicate to use as the capture antigen for the ELISA, PAO1 bacterial suspension was autoclaved for 15 min at 121°C and 15 kPa. The suspension was sonicated on ice three times with a Branson Sonifier® S250D digital sonicator using a fine point probe (Branson Ultrasonics Corp., Danbury, CT, USA) before the cellular debris was pelleted at 4°C and 3000× g for 10 min. The supernatant was then aliquotted and stored at –80°C. A bicinchoninic acid assay was used to determine the protein concentration. The concentration of the unknown was calculated against a BSA standard curve.

#### Preparation of P. aeruginosa IgG and IgM positive and negative sera

Heat-killed bacteria were diluted to the equivalent of 2×10^9^ CFU/mL and 50 µL (1×10^8^ CFU equivalents) were injected intraperitoneally into male BALB/c mice. Anti-*P. aeruginosa* IgM-positive sera was obtained after 8 days, and anti-*P. aeruginosa* IgG-positive was obtained after 24 days with repeat injections on day 8 and day 16. Negative control serum was collected from unimmunised mice. The positive sera were arbitrarily defined as containing 1×10^6^ U/mL of anti-*P. aeruginosa* antibody.

#### Anti-P. aeruginosa IgG and IgM ELISA

Ninety-six well Polysorp microtiter plates (Nunc, Rochester, NY, USA) were coated with 100 µL/well of *P. aeruginosa* sonicate diluted to 10 µg/mL in coating buffer (1.59 g/L Na_2_CO_3_ and 2.94 g/L NaHCO_3_ in diH_2_O pH 9.6). The plates were sealed and incubated overnight at 4°C. The plates were then washed three times in Dulbecco’s modified phosphate buffered saline (DPBS) supplemented with 0.05% v/v Tween-20 then blocked with reagent diluent (DPBS with 5% skim milk powder, prepared on the day). All samples were prepared in reagent diluents and a standard curve was generated using the positive control serum.

After blocking, the plate was washed three times with washing buffer and then 100 µL/well of the samples added in duplicate to the plate, which was sealed and incubated at room temperature for 2 h. Following this incubation, the plate was washed with DPBS-Tween and 100 µL/well of the diluted detection antibodies added. The detection antibodies were diluted in reagent diluent to the following concentrations: biotinylated anti-mouse IgG1 heavy chain (1 µg/mL); biotinylated anti-mouse IgG2a heavy chain (1 µg/mL); and biotinylated anti-mouse IgM heavy chain (0.25 µg/mL) (all AbD Serotec, Oxford, UK) and allowed to incubate at room temperature for at least 10 min. The plate was sealed and incubated at room temperature for 2 h.

The plate was washed with DPBS-Tween and 100 µL/well of 0.2 µg/mL Streptavidin-HRP (Amersham Biosciences) diluted in 1% w/v BSA in DPBS was added, the plate sealed and incubated at room temperature for 1 h. After the incubation, the plate was washed with DPBS-Tween and 100 µL/well of 3,3,5,5′-tetramethylbenzidine diluted to 110 µg/mL in citrate acetate (pH 9.6) supplemented with 0.2% v/v H_2_O_2_, and the colour left to develop for 5 min. The development was stopped by the addition of 100 µL/well of 0.16 M H_2_SO_4_. The absorbance was then read at 450 nm with a reference filter of 520 nm on a BioRad microplate reader and the software package Microplate Manager v 5.2.1 was used to calculate the standard curve. The least dilute sample with an absorbance reading that fit on the linear portion of the curve was used to calculate the antibody concentration of the sample.

#### Statistical analysis

All statistical analysis was performed using GraphPad Prism v 5. Distribution of all data was tested for normality using the Kolmogorov–Smirnov test. Data sets that were normally distributed are expressed as mean ± standard deviation, and differences between groups were evaluated using *t-*test or ANOVA. Data sets (antibody titers) that were not normally distributed are presented as median with individual points. Differences between groups were evaluated using the Mann–Whitney *U* test.

## Results

### Phenotypic Characterisation of PAO1Δ*lasIR*, PAO1Δ*rhlIR* and PAO1Δ*lasIRrhlIR* Deletion Mutants

To re-examine the role of quorum sensing in infection, we generated an unmarked deletion mutant of PAO1 that lacks both the *las* and *rhl* quorum sensing systems. We also further minimized any chance of other mutations arising by limiting its subculturing prior to characterization in various phenotypic assays and mouse models of infection. Because of the hierarchical nature of the *las* and *rhl* quorum sensing systems the PAO1Δ*rhlIR* mutant was created first as a *lasIR* knockout would also be deficient in many of the phenotypes that we used to confirm the *rhlIR* knockout.

**Figure 1:**
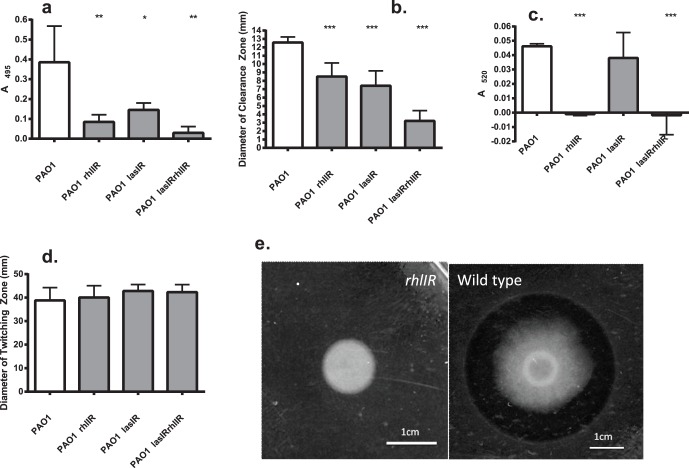
Phenotypic characteristics of QS mutants. Elastase production (a), protease production (b) pyocyanin production (c) twitching motility (d) rhamnolipid production (e) of QS mutants compared with parental PAO1. Rhamnolipid production only tested for *ΔrhlIR* mutant. All results represented as means ± SD of at least triplicate determinations. Differences between groups assessed with unpaired *t-*test; **P<0.05; **P<0.01; ***P<0.001.

An unmarked PAO1Δ*rhlIR* deletion mutant was generated and a number of phenotypic assays were performed to confirm that this strain displayed phenotypes consistent with previously published observations for mutants of *rhlI* and *rhlR* and to confirm that it had not acquired secondary mutations that result in defective twitching motility. As expected, our PAO1Δ*rhlIR* mutant produced reduced levels of elastase (Fig. 1a) and protease (Fig. 1b) relative to PAO1, did not produce rhamnolipid (Fig. 1e), produced barely detectable levels of pyocyanin (Fig. 1c), and retained wild-type twitching motility (Fig. 1d).

After verification of the PAO1Δ*rhlIR* mutant phenotypes, PAO1Δ*lasIR* and PAO1Δ*lasIRrhlIR* deletion mutants were created. These mutants were assayed to verify that they had phenotypes consistent with published observations and that they had not acquired secondary mutations that result in defective twitching motility. PAO1Δ*lasIR* showed reduced levels of elastase production whereas the PAO1Δ*lasIRrhlIR* quadruple knockouts produced barely detectable levels of elastase (Fig. 1a). Protease production by PAO1Δ*lasIR* was significantly reduced compared with the wild-type control, and was even lower in the quadruple knockout PAO1Δ*lasIRrhlIR* (Fig. 1b). PAO1Δ*lasIR* did not show a significant defect in pyocyanin production whereas PAO1Δ*lasIRrhlIR* produced barely detectable levels of pyocyanin (Fig. 1c). Importantly, both PAO1Δ*lasIR* and PAO1Δ*lasIRrhlIR* retained wild type levels of twitching motility (Fig. 1d).

**Figure 2:**
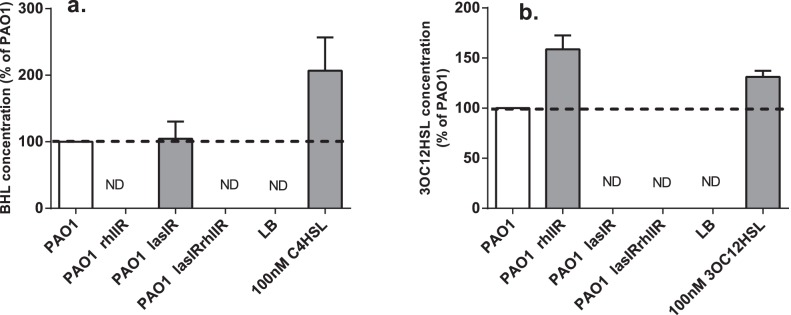
Production of C4HSL and 3OC12HSL by QS mutants compared with PAO1. The capacity of all mutants to produce C4HSL (a) and 3OC12HSL (b) is presented. Because of interassay variability, all results are presented as percent of production by wild type PAO1. Results for 100 µM C4HSL (a) or 3OC12HSL (b) included as positive controls. Values represent mean ± SD of at least triplicate determinations. LB: LB broth (negative control). ND: not detected.

All mutants were also tested to determine the levels of production of C4HSL or 3OC12HSL in the culture supernatant using bioluminescence reporters specific for C4HSL [23] and 3OC12HSL [24]. The concentration of AHLs produced was calculated using a standard curve of synthetic AHL, but because the interassay variation was large, the results are presented as a percentage of the wild-type (Fig. 2a, b). The *rhlIR* mutant produced no detectable C4HSL and higher levels of 3OC12HSL than wild type PAO1; the *lasIR* mutant produced similar levels of C4HSL to wild type and no detectable 3OC12HSL, and the *lasIRrhlIR* mutant produced no detectable C4HSL or 3OC12HSL.

### Characteristics of Lung Infection in BALB/c Mice

**Figure 3:**
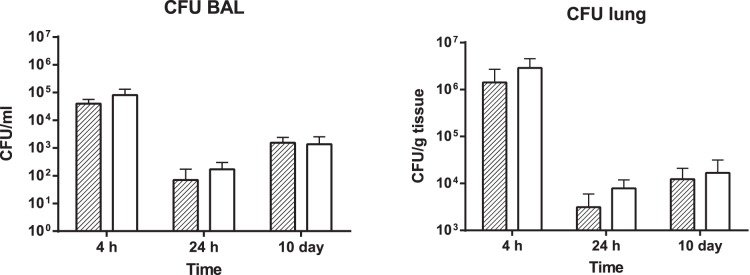
Bacterial recovery from lungs of mice infected with PAO1 or PAO1Δ*lasIRrhlIR*. Bronchoalveolar lavage and lung tissue was collected from groups of five mice at 4 h, 24 h and 10 d after intratracheal infection with 1×10^6^ CFU of either PAO1 (hatched bars) or PAO1Δ*lasIRrhlI* (QSneg, open bars). Lung tissue was homogenised, serial dilutions made in PBS and cultured on LBA to estimate bacterial numbers in lung tissue. An aliquot of BAL fluid was also serially diluted in PBS and dilutions plated on LBA to estimate bacterial numbers in BAL (airways). Results expressed as mean ± SD for five mice at each point. There were no significant differences in numbers of PAO1 and PAO1Δ*lasIRrhlIR* recovered at any time point.

To evaluate the effects of knocking out both *lasIR* and *rhlIR* on the course of an infection, groups of BALB/c mice were infected with either PAO1 or PAO1Δ*lasIRrhlIR,* and followed for 10 days. Mice were euthanized at 4 h, 24 h and 10 days after infection, and bacterial numbers, lung levels of inflammatory leukocytes, and lung expression of mRNA for inflammatory cytokines TNFα, IL-1β and mKC (IL-8 analogue) were evaluated. The bacterial numbers recovered from lung tissue and BAL at each time point are shown in Fig. 3, and indicate that at each time point there were no significant differences between wild type PAO1 and the PAO1Δ*lasIRrhlIR* mutant, although there was a trend to recovery of higher numbers of PAO1Δ*lasIRrhlIR* than PAO1 at 4 h and 24 h. Low levels of infection with both strains were maintained out to 10 days.

**Figure 4:**
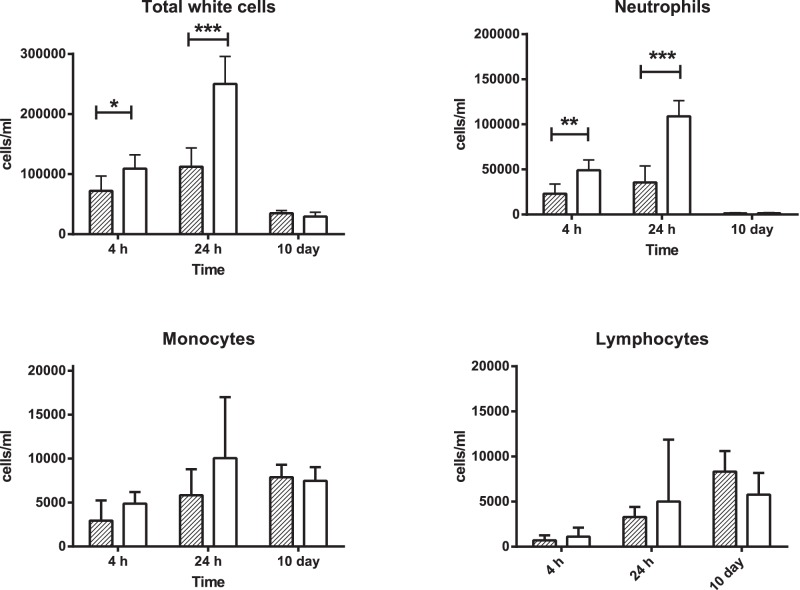
Leukocyte infiltrate in bronchoalveolar lavage (BAL) fluid from lungs of mice infected with PAO1 or PAO1Δ*lasIRrhlIR*. BAL fluid was collected from mice at 4 h, 24 h and 10 d after intratracheal infection with 1×10^6^ CFU of either PAO1 (hatched bars) or PAO1Δ*lasIRrhlIR* (QSneg, open bars). An aliquot of fluid from each mouse was centrifuged in a cytospin, and slides stained with DiffQuik for differential counting. A minimum of 200 cells per slide were counted. Results expressed as mean ± SD for individual BAL fluids from five mice per group at each time point. Differences between groups analysed with an unpaired *t* test. *P<0.05, **P<0.01, ***P<0.001.

We also investigated leukocyte infiltration into the lung by examining leukocytes in BAL at all time points (Fig. 4). At 4 h and 24 h, the infiltrate was dominated by neutrophils (80–90%), whereas at day 10, neutrophils constituted only about 10% of infiltrating cells, with monocytes and other cells (mostly lymphocytes) both representing approximately 45% of cells. The only significant differences between mice inoculated with PAO1 and PAO1Δ*lasIRrhlIR* were in total white cells and neutrophils at 4 h and 24 h post infection, where neutrophil numbers (and hence total white cell counts) were significantly higher in mice infected with PAO1Δ*lasIRrhlIR* (*p<*0.01, unpaired *t* test).

**Figure 5:**
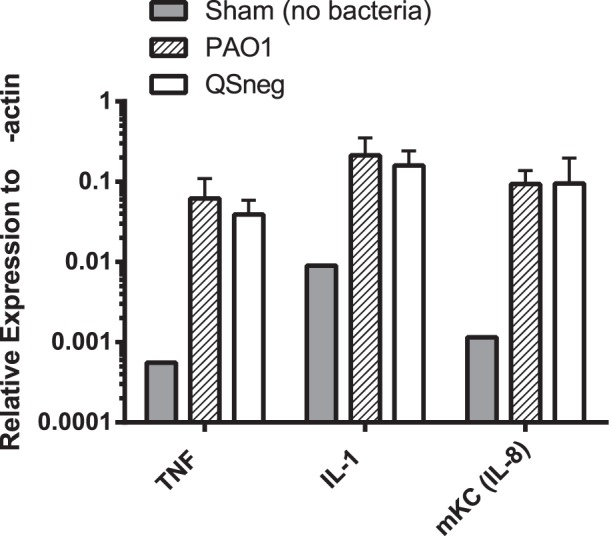
Expression of cytokine mRNA in lung tissue of mice 4 h after infection with PAO1 or PAO1Δ*lasIRrhlIR*. Lung tissue from mice infected with 1×10^6^ CFU of either PAO1 (hatched bars) or PAO1Δ*lasIRrhlI* (QSneg, open bars) was processed, total RNA extracted and cDNA prepared. Expression of cytokine genes was assessed in RT-qPCR using β-actin as the reference gene. All results are expressed as gene expression relative to β-actin, and are presented as the mean ± SD of five mice per group. A control group of lungs from mice that underwent anaesthesia and intratracheal infusion of PBS without bacteria was included (grey bars). Expression of all cytokines was significantly higher in mice infected with bacteria than in sham-infected mice (*P*<0.01, all groups, ANOVA) but there were no significant differences between mice infected with PAO1 or PAO1Δ*lasIRrhlIR* (QSneg).

We used RT-qPCR to investigate the level of expression of three cytokines characteristic of acute inflammation, TNFα, IL1β and mKC, a mouse analogue of human IL-8, at 24 h post infection. The results, shown in Fig. 5, demonstrate that there was no significant difference in the level of expression of any of these cytokines between mice infected with PAO1 and those infected with PAO1Δ*lasIRrhlIR*.

**Figure 6:**
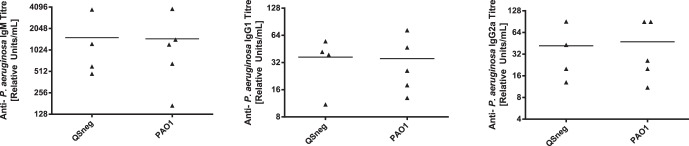
Anti-*Pseudomonas* antibody production by mice infected with PAO1 or PAO1Δ*lasIRrhlIR*. Mice were infected intratracheally with 1×10^3^ CFU of PAO1 or PAO1Δ*lasIRrhlIR* in an 0.5% agar slurry, and bled on day 22. Sera were tested in ELISA against antigen prepared from whole killed PAO1, using isotype-specific detection antibodies for mouse IgM, IgG1, and IgG2a. Results are expressed as individual titers with a line representing the median values. There were no significant differences (Mann–Whitney *U* test) between mice infected with PAO1 or PAO1Δ*lasIRrhlIR* (QSneg).

Lastly, in a different set of experiments, mice were infected with PAO1 or PAO1Δ*lasIRrhlIR* embedded in agar slurry, euthanized at 22 days after initial infection and after a second dose of bacteria at day 21, and serum levels of *P. aeruginosa*-specific IgM, IgG1 and IgG2a measured by ELISA. Figure 6 shows that there was no significant difference in the titres of any of the isotypes between mice infected with PAO1 and those infected with PAO1Δ*lasIRrhlIR.*

## Discussion

The literature contains numerous examples of animal models of *P. aeruginosa* infection. In many of these, a comparison of a wild type (usually PAO1) and a variety of QS-deficient strains suggest that QS-controlled virulence factors are important in the pathogenesis of infection, and that most QS mutants are less virulent than wild type *P. aeruginosa.* It is unquestionable that QS controlled biofilm formation and virulence factor production plays an important role in bacterial virulence, particularly in clinical settings such as cystic fibrosis (CF) (e.g. [7]). However, because many QS mutants are known to acquire other mutations, particularly in twitching motility, which could affect infection and dissemination, we decided to create a rigorously designed quadruple knockout, and further minimize any chance of other mutations arising by limiting its culturing. Such a knockout would allow the separation of effects of bacterial adherence and infectivity from the effects of QS-controlled virulence.

Creation of the PAO1Δ*lasIRrhlIR* was a multi-step process. A PAO1Δ*rhlIR* mutant was created first because of the hierarchical nature of the *las* and *rhl* quorum sensing systems: a *lasIR* knockout would be deficient in many of the phenotypes used to confirm a *rhlIR* knockout, making the phenotypic confirmation of a *rhlIR* knockout on a PAO1Δ*lasIR* background more difficult. However, creating the *rhlIR* knockout first enabled us to confirm that its phenotype was as expected, with loss of pyocyanin production being the key phenotype change. The deletion of the *lasI* and *lasR* genes also resulted in the deletion of *rsaL*, but we predicted that an *rsaL* deletion would not affect the mutant significantly because its product is a *lasI* repressor, and this was supported by the phenotypic studies on the PAO1Δ*lasIRrhlIR* and PAO1Δ*lasIR* mutants generated. The characteristics of the PAO1Δ*lasIRrhlIR* knockouts clearly demonstrate that they are unable to produce a number of key QS-regulated virulence factors, and cannot respond to exogenous AHLs, but that they retain full twitching capability.

The results of the studies in the mouse model of lung infection suggest that there is no significant difference between the persistence of PAO1 and PAO1Δ*lasIRrhlIR* in mouse lungs, or in their ability to induce an inflammatory response in the host, although there is a suggestion from the neutrophil counts that PAO1Δ*lasIRrhlIR* actually induces a stronger inflammatory infiltrate than the parental PAO1, and the trend to higher bacterial numbers recovered in PAO1Δ*lasIRrhlIR* infected mice is consistent with the slightly faster growth rate of this strain (results not shown). Wu *et al.* also observed a stronger inflammatory response to a PAO1 *lasIrhlI* knockout strain (PAO1-JP2) in a rat model, but unlike in our study, this was accompanied by a more rapid clearance of bacteria [11]. However, as PAO1-JP2 is known to be deficient in twitching motility [14], this is likely to affect its ability to colonise lungs and hence its ability to persist. However, other studies in rats and mice have also suggested that quorum sensing-defective mutants are cleared more effectively than wild type bacteria [11], [36]. For example, a study using a neonatal BALB/c model of infection reported that a PAO1Δ*lasR* mutant was virtually avirulent, causing no mortality, no evidence of lung pathology and only 15% of mice showed evidence of bacterial replication *in vivo* [37], although the authors found that PAO1Δ*lasR* were still recovered in the lungs 24 h post infection in numbers similar to PAO1, indicating that although the bacteria could persist they did not cause pathology [37]. In our study, the bacterial numbers in PAO1Δ*lasIRrhlIR*-infected and wild type PAO1-infected mice were similar at each time point, with low numbers persisting out to at least 10 days after infection. Analysis of the cytokine mRNA in the lungs indicates that there is little difference in the ability of the two strains to stimulate effective proinflammatory cytokine responses, although analysis of the PMN recruitment to the lung indicates that PAO1Δ*lasIRrhlIR* is more effective at stimulating innate immunity.

An explanation for some of the differences between our results and those of others may be that, although PAO1 is the laboratory standard for *P. aeruginosa*, there is a great deal of genetic diversity between laboratories in the PAO1 strains used [13]. For example, twitching motility is one of the phenotypes commonly lost during ongoing laboratory culture, because of the development of point mutations in other regulators such as *vfr* [12]. For example, the PAO1 *lasIrhlI* knockout strain (PAO1-JP2) used in many studies has been reported to lack twitching motility [14] and this can reduce the ability of the bacteria to colonize mammalian epithelia. Thus, some of the reported reduced virulence of QS mutant *P. aeruginosa* may be the consequence of impaired colonization by twitching-deficient bacteria. Care was taken in the creation of the PAO1Δ*lasIRrhlIR* mutant used in this study to ensure that such secondary mutations had not occurred, increasing confidence that the results from this study were in fact a reflection of the differences in quorum sensing ability of PAO1Δ*lasIRrhIR* and wild type PAO1, and not of other mutations in PAO1.

High antibody titres against *P. aeruginosa* have been associated with decreases in lung function and poorer outcomes of infection [38], [39]. There were detectable titres of anti-*P*. *aeruginosa* IgM antibodies at 10 days post lung infection in the acute infection model, but no detectable IgG1 or IgG2a, and no difference between PAO1-infected mice and those infected with PAO1Δ*lasIRrhIR* (results not shown). To evaluate later immune responses, we generated a more chronic infection by giving mice *P. aeruginosa* in an agar slurry, which delays bacterial clearance, and collecting serum at day 22 after an acute bacterial challenge on day 21. *P. aeruginosa*-specific IgM, IgG1 and IgG2a were all easily detectable at this time point, but there was no difference in the titers of any of the isotypes between PAO1ΔlasIRrhlIR-infected and PAO1-infected mice. This is in contrast to a previous report using a rat model of infection suggesting that the IgG1 response was higher in rats infected with PAO1 compared with rats infected with a QS signal-deficient mutant (PAO1Δ*lasIrhlI*) [40]. As IgG1 is associated with prohumoral Th2 type responses in mice, this implied that QS-deficient mutants generated a more Th2-biased response, but we saw no such difference. The difference between our results and those reported by Wu *et al*. could be due to species variation, because of the differences in persistence between PAO1 and PAO1Δ*lasIrhlI* observed in Wu *et al*.’s study, where the mutant was cleared more effectively than the wild type, or could be related to differences in the bacterial strains other than those defined.

The mouse model used in this study was an acute lung infection, and our results do not exclude the likelihood that QS-mediated induction of biofilm formation and virulence factor production play important roles in chronic infections. It would be of interest to repeat these studies in models of chronic infection, particularly immune deficiency or cystic fibrosis, which in humans, results in increased susceptibility to *P. aeruginosa* infection. It remains entirely possible that QS mutants may be less virulent in such situations than wild type *P. aeruginosa,* for a combination of reasons related to both defects in biofilm formation and virulence factor production and to host factors and the characteristics of the inflammatory and immune responses to infection, which are known to be aberrant in people with cystic fibrosis (CF) [41]. In this context, although it is clear that *P. aeruginosa* exists in biofilms in CF lung [42], [43] and the bacterial populations present in CF lung are heterogeneous and always include some QS-competent bacteria [42], it is relevant to note that it has been reported that QS-deficient mutants arise spontaneously after some years in people with CF with chronic *P. aeruginosa* lung infection [7]. The most frequently observed mutation in the genes of the QS systems is in *lasR* ¸ which could suggest that while QS systems are important for initiating and maintaining chronic infection, “social cheating” by subpopulations of QS-deficient *P. aeruginosa* could confer a survival advantage in the environment of the chronically-infected and damaged CF lung.

In conclusion, our results suggest that careful control of culture and propagation of QS deficient PAO1 derivatives to prevent additional mutations and the loss of such important characteristics as twitching motility may eliminate a significant proportion of the previously observed differences in infectivity and persistence of QS-deficient bacteria and wild-type *P. aeruginosa* in animal models, and allow a more precise evaluation of the importance of QS and QS-regulated gene expression in bacterial virulence and pathogenesis of infection.
